# Methyl 4-hydr­oxy-2-propyl-2*H*-1,2-benzothia­zine-3-carboxyl­ate 1,1-dioxide

**DOI:** 10.1107/S1600536809046236

**Published:** 2009-11-07

**Authors:** Muhammad Nadeem Arshad, Muhammad Zia-ur-Rehman, Islam Ullah Khan

**Affiliations:** a Department of Chemistry, Government College University, Lahore 54000, Pakistan; b Applied Chemistry Research Centre, PCSIR Laboratories Complex, Lahore 54600, Pakistan

## Abstract

In the title compound, C_13_H_15_NO_5_S, the thia­zine ring adopts a distorted half-chair conformation. The enolic H atom is involved in an intra­molecular O—H⋯O hydrogen bond, forming a six-membered ring. In the crystal, mol­ecules are linked through weak inter­molecular C—H⋯O hydrogen bonds, resulting in zigzag chains lying along the *c* axis.

## Related literature

For the syntheses of related compounds, see: Bihovsky *et al.* (2004 ▶); Braun (1923 ▶); Lombardino *et al.* (1971 ▶); Zia-ur-Rehman *et al.* (2005 ▶, 2009 ▶). For the biological activity of benzothia­zines, see: Turck *et al.* (1996 ▶); Zia-ur-Rehman *et al.* (2006 ▶). For related structures, see: Fabiola *et al.* (1998 ▶); Zia-ur-Rehman *et al.* (2007 ▶).
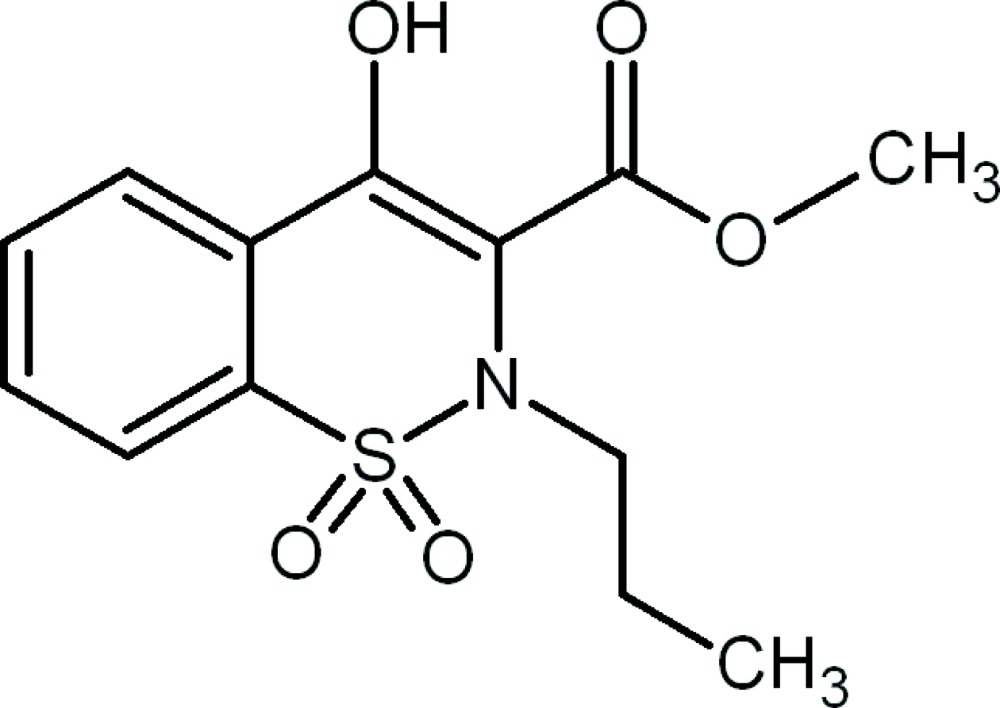


## Experimental

### 

#### Crystal data

C_13_H_15_NO_5_S
*M* *_r_* = 297.32
Orthorhombic, 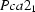

*a* = 12.4398 (6) Å
*b* = 8.7538 (5) Å
*c* = 12.7288 (7) Å
*V* = 1386.11 (13) Å^3^
*Z* = 4
Mo *K*α radiation
μ = 0.25 mm^−1^
*T* = 296 K
0.39 × 0.36 × 0.11 mm

#### Data collection

Bruker APEXII CCD area-detector diffractometer
Absorption correction: multi-scan (**SADABS**; Sheldrick, 1996 ▶) *T*_min_ = 0.908, *T*_max_ = 0.973
8843 measured reflections
3252 independent reflections
2540 reflections with *I* > 2σ(*I*)
*R*_int_ = 0.028

#### Refinement

*R*[*F* ^2^ > 2σ(*F* ^2^)] = 0.036
*wR*(*F* ^2^) = 0.094
*S* = 1.03
3252 reflections
184 parameters
1 restraint
H-atom parameters constrained
Δρ_max_ = 0.16 e Å^−3^
Δρ_min_ = −0.22 e Å^−3^
Absolute structure: Flack (1983 ▶), 1451 Friedel pairs
Flack parameter: −0.08 (8)

### 

Data collection: *APEX2* (Bruker, 2007 ▶); cell refinement: *SAINT* (Bruker, 2007 ▶); data reduction: *SAINT*; program(s) used to solve structure: *SHELXS97* (Sheldrick, 2008 ▶); program(s) used to refine structure: *SHELXL97* (Sheldrick, 2008 ▶); molecular graphics: *PLATON* (Spek, 2009 ▶) and *Mercury* (Macrae *et al.*, 2006 ▶); software used to prepare material for publication: *WinGX* (Farrugia, 1999 ▶) and *PLATON*.

## Supplementary Material

Crystal structure: contains datablocks I, global. DOI: 10.1107/S1600536809046236/is2482sup1.cif

Structure factors: contains datablocks I. DOI: 10.1107/S1600536809046236/is2482Isup2.hkl

Additional supplementary materials:  crystallographic information; 3D view; checkCIF report

## Figures and Tables

**Table 1:** Hydrogen-bond geometry (Å, °)

*D*—H⋯*A*	*D*—H	H⋯*A*	*D*⋯*A*	*D*—H⋯*A*
O3—H3*A*⋯O4	0.82	1.84	2.558 (2)	145
C3—H3⋯O2^i^	0.93	2.48	3.358 (3)	158

Symmetry code: (i) 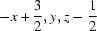.

**Fig. 1.**
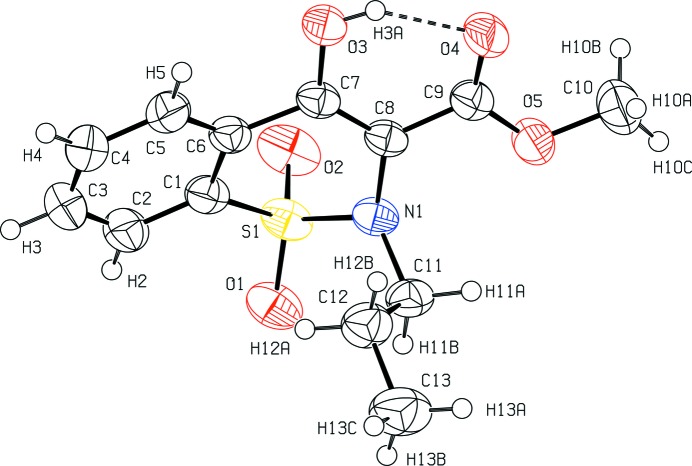
The molecular structure of (I), with displacement ellipsoids at the 50% probability level.

**Fig. 2.**
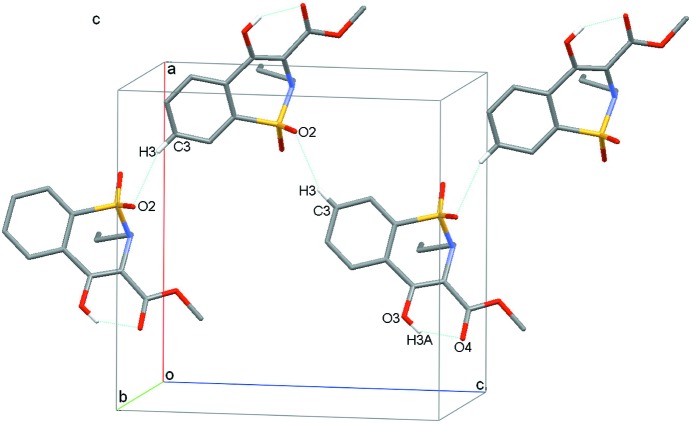
Perspective view of the crystal packing showing C—H···O hydrogen-bonded interactions (dashed lines). H atoms not involved in hydrogen bonding have been omitted for clarity.

## supplementary crystallographic information

### Comment

Benzothiazine1,1-dioxides are known to possess a versatile range of biological activities and have been synthesized continuously since the very first synthesis in 1923 (Braun, 1923). Among these, Piroxicam (Lombardino *et al.*, 1971; Zia-ur-Rehman *et al.*, 2005), and Meloxicam (Turck *et al.*, 1996) are familiar for their analgesic and anti-inflammatory activities and are being used world wide as non-steroidal anti-inflammatory drugs (NSAIDs). Few of its derivatives are also known as potent calpain I inhibitors (Bihovsky *et al.*, 2004), while benzothiaine-3-yl-quinazolin-4-ones showed marked activity against *Bacillus subtilis *(Zia-ur-Rehman *et al.*, 2006). As part of a research program synthesizing various bioactive benzothiazines (Zia-ur-Rehman *et al.*, 2005, 2006, 2009), we herein report the crystal structure of the title compound, (I).

In the molecule of the title compound (Fig. 1), the thiazine ring exhibits a distorted half-chair conformation with S1/C1/C6/C7 atoms lying in a plane and N1 showing significant departure from the plane due to its pyramidal geometry projecting the propyl group approximately perpendicular to the ring. Like other 1,2-benzothiazine 1,1-dioxide derivatives (Fabiola *et al.*, 1998; Zia-ur-Rehman *et al.*, 2007), the enolic hydrogen on O3 is involved in intramolecular hydrogen bonding (Table1). Also, C7—C8 bond length [1.346 (3) Å] (very close to normal C—C bond 1.36 Å) indicates a partial double-bond character indicating the dominance of enolic form in the molecule. The C1—S1 bond distance [1.759 (3) Å] is as expected for typical C(*sp*2)—S bond (1.751 Å). Each molecule is linked to neighbouring molecules *via *weaker C—H···O=S interactions giving rise to zigzag chains along the *c* axis (Fig. 2).

### Experimental

Propyl iodide (5.10 g, 30.0 mmol) was added drop wise to the mixture of methyl 4-hydroxy-2*H*-1,2-benzothiazine-3-carboxylate-1,1-dioxide (3.83 g, 15.0 mmol), anhydrous potassium carbonate (1.68 g, 30.0 mmol) and dimethylformamide (20.0 ml) in a round bottom flask. Contents were stirred at room temperature for 7 h under nitrogen atmosphere and poured over ice cooled water (300 ml) resulting in an immediate formation of a white solid, which was filtered and washed with cold water. Crystallization from methanol yielded pure compound.

### Refinement

All hydrogen atoms were identified in the difference map and subsequently fixed in ideal positions and treated as riding on their parent atoms. In the case of the methyl and hydroxyl H atoms the torsion angles were freely refined. The following distances were used: C—H = 0.98 Å for methyl, C—H = 0.95 Å for aromatic and O—H = 0.84 Å for hydroxyl. *U*_iso_(H) was set to 1.2*U*_eq_ of the parent atoms or 1.5*U*_eq_ for methyl groups.

### Crystal data

**Table:**

C_13_H_15_NO_5_S	*F*(000) = 624
*M**_r_* = 297.32	*D*_x_ = 1.425 Mg m^−^^3^
Orthorhombic, *P**c**a*2_1_	Mo *K*α radiation, λ = 0.71073 Å
Hall symbol: P 2c -2ac	Cell parameters from 2980 reflections
*a* = 12.4398 (6) Å	θ = 2.3–25.3°
*b* = 8.7538 (5) Å	µ = 0.25 mm^−^^1^
*c* = 12.7288 (7) Å	*T* = 296 K
*V* = 1386.11 (13) Å^3^	Rods, yellow
*Z* = 4	0.39 × 0.36 × 0.11 mm

### Data collection

**Table:**

Bruker APEXII CCD area-detector diffractometer	3252 independent reflections
Radiation source: fine-focus sealed tube	2540 reflections with *I* > 2σ(*I*)
graphite	*R*_int_ = 0.028
φ and ω scans	θ_max_ = 28.3°, θ_min_ = 2.9°
Absorption correction: multi-scan (*SADABS*; Sheldrick, 1996)	*h* = −16→10
*T*_min_ = 0.908, *T*_max_ = 0.973	*k* = −11→11
8843 measured reflections	*l* = −16→15

### Refinement

**Table:**

Refinement on *F*^2^	Secondary atom site location: difference Fourier map
Least-squares matrix: full	Hydrogen site location: inferred from neighbouring sites
*R*[*F*^2^ > 2σ(*F*^2^)] = 0.036	H-atom parameters constrained
*wR*(*F*^2^) = 0.094	*w* = 1/[σ^2^(*F*_o_^2^) + (0.0496*P*)^2^] where *P* = (*F*_o_^2^ + 2*F*_c_^2^)/3
*S* = 1.03	(Δ/σ)_max_ < 0.001
3252 reflections	Δρ_max_ = 0.16 e Å^−^^3^
184 parameters	Δρ_min_ = −0.22 e Å^−^^3^
1 restraint	Absolute structure: Flack (1983), 1451 Friedel pairs
Primary atom site location: structure-invariant direct methods	Flack parameter: −0.08 (8)

### Special details

**Table:**

Geometry. All e.s.d.'s (except the e.s.d. in the dihedral angle between two l.s. planes) are estimated using the full covariance matrix. The cell e.s.d.'s are taken into account individually in the estimation of e.s.d.'s in distances, angles and torsion angles; correlations between e.s.d.'s in cell parameters are only used when they are defined by crystal symmetry. An approximate (isotropic) treatment of cell e.s.d.'s is used for estimating e.s.d.'s involving l.s. planes.
Refinement. Refinement of *F*^2^ against ALL reflections. The weighted *R*-factor *wR* and goodness of fit *S* are based on *F*^2^, conventional *R*-factors *R* are based on *F*, with *F* set to zero for negative *F*^2^. The threshold expression of *F*^2^ > σ(*F*^2^) is used only for calculating *R*-factors(gt) *etc*. and is not relevant to the choice of reflections for refinement. *R*-factors based on *F*^2^ are statistically about twice as large as those based on *F*, and *R*- factors based on ALL data will be even larger.

### Fractional atomic coordinates and isotropic or equivalent isotropic displacement parameters (Å^2^)

**Table:**

	*x*	*y*	*z*	*U*_iso_*/*U*_eq_	
C1	0.58950 (17)	0.7963 (3)	0.8319 (2)	0.0442 (5)	
C2	0.66903 (18)	0.8114 (3)	0.7577 (2)	0.0565 (6)	
H2	0.7391	0.7816	0.7726	0.068*	
C3	0.6434 (2)	0.8714 (3)	0.6609 (3)	0.0703 (8)	
H3	0.6967	0.8829	0.6103	0.084*	
C4	0.5396 (2)	0.9145 (3)	0.6385 (2)	0.0622 (7)	
H4	0.5230	0.9536	0.5725	0.075*	
C5	0.45981 (19)	0.9001 (3)	0.71319 (19)	0.0509 (6)	
H5	0.3900	0.9308	0.6976	0.061*	
C6	0.48305 (15)	0.8403 (3)	0.81103 (17)	0.0407 (5)	
C7	0.39979 (15)	0.8187 (3)	0.89165 (18)	0.0398 (5)	
C8	0.40966 (15)	0.7206 (2)	0.97246 (19)	0.0389 (5)	
C9	0.32273 (17)	0.7043 (3)	1.04807 (18)	0.0443 (5)	
C10	0.2568 (3)	0.5866 (3)	1.2006 (3)	0.0755 (8)	
H10A	0.1955	0.5455	1.1645	0.113*	
H10B	0.2378	0.6825	1.2322	0.113*	
H10C	0.2794	0.5165	1.2542	0.113*	
C11	0.50112 (17)	0.4660 (3)	0.9658 (2)	0.0493 (5)	
H11A	0.4465	0.4214	1.0107	0.059*	
H11B	0.5695	0.4204	0.9848	0.059*	
C12	0.4761 (2)	0.4257 (3)	0.8530 (2)	0.0589 (7)	
H12A	0.5279	0.4748	0.8072	0.071*	
H12B	0.4052	0.4639	0.8350	0.071*	
C13	0.4794 (3)	0.2548 (3)	0.8355 (3)	0.0812 (10)	
H13A	0.4297	0.2057	0.8823	0.122*	
H13B	0.5507	0.2178	0.8490	0.122*	
H13C	0.4600	0.2325	0.7641	0.122*	
N1	0.50637 (13)	0.6325 (2)	0.98684 (13)	0.0423 (5)	
O1	0.70303 (11)	0.6188 (2)	0.95391 (16)	0.0633 (5)	
O2	0.62415 (13)	0.8534 (2)	1.02728 (17)	0.0676 (6)	
O3	0.31275 (12)	0.90699 (18)	0.87688 (13)	0.0532 (4)	
H3A	0.2671	0.8861	0.9211	0.080*	
O4	0.23788 (13)	0.7728 (2)	1.03972 (15)	0.0586 (5)	
O5	0.34308 (13)	0.60963 (19)	1.12699 (13)	0.0547 (4)	
S1	0.61649 (4)	0.72551 (7)	0.95848 (6)	0.04848 (16)	

### Atomic displacement parameters (Å^2^)

**Table:**

	*U*^11^	*U*^22^	*U*^33^	*U*^12^	*U*^13^	*U*^23^
C1	0.0361 (10)	0.0384 (12)	0.0580 (13)	−0.0019 (9)	0.0015 (10)	−0.0081 (10)
C2	0.0407 (13)	0.0523 (15)	0.0765 (17)	−0.0002 (11)	0.0107 (12)	−0.0006 (13)
C3	0.0666 (18)	0.0632 (18)	0.081 (2)	0.0040 (15)	0.0342 (15)	0.0044 (15)
C4	0.0709 (17)	0.0561 (17)	0.0595 (16)	0.0069 (13)	0.0155 (14)	0.0104 (13)
C5	0.0502 (13)	0.0437 (14)	0.0587 (15)	0.0032 (10)	0.0017 (11)	0.0047 (11)
C6	0.0369 (10)	0.0354 (12)	0.0499 (13)	−0.0005 (9)	0.0016 (9)	−0.0056 (10)
C7	0.0322 (10)	0.0402 (12)	0.0471 (12)	0.0021 (8)	−0.0027 (9)	−0.0072 (9)
C8	0.0315 (8)	0.0412 (11)	0.0439 (14)	0.0042 (8)	−0.0029 (9)	−0.0057 (10)
C9	0.0403 (12)	0.0447 (12)	0.0480 (12)	0.0016 (10)	0.0004 (10)	−0.0040 (11)
C10	0.0678 (16)	0.095 (2)	0.0642 (17)	0.0176 (16)	0.0203 (13)	0.0219 (18)
C11	0.0443 (10)	0.0444 (12)	0.0592 (13)	0.0112 (9)	−0.0031 (11)	0.0016 (13)
C12	0.0590 (14)	0.0499 (16)	0.0677 (17)	−0.0024 (11)	−0.0011 (13)	−0.0091 (12)
C13	0.077 (2)	0.059 (2)	0.107 (3)	−0.0003 (14)	0.0020 (18)	−0.0210 (19)
N1	0.0350 (9)	0.0465 (11)	0.0455 (12)	0.0077 (7)	−0.0061 (7)	−0.0044 (8)
O1	0.0354 (7)	0.0806 (12)	0.0740 (11)	0.0161 (7)	−0.0059 (9)	−0.0045 (11)
O2	0.0468 (10)	0.0802 (14)	0.0760 (12)	−0.0004 (8)	−0.0149 (8)	−0.0329 (11)
O3	0.0378 (8)	0.0548 (10)	0.0669 (11)	0.0137 (7)	0.0065 (7)	0.0114 (8)
O4	0.0413 (9)	0.0678 (11)	0.0669 (11)	0.0144 (8)	0.0100 (8)	0.0109 (9)
O5	0.0484 (9)	0.0677 (11)	0.0480 (9)	0.0105 (8)	0.0071 (7)	0.0092 (8)
S1	0.0309 (2)	0.0583 (3)	0.0562 (3)	0.0046 (2)	−0.0086 (3)	−0.0146 (3)

### Geometric parameters (Å, °)

**Table:**

C1—C2	1.374 (3)	C10—O5	1.439 (3)
C1—C6	1.404 (3)	C10—H10A	0.9600
C1—S1	1.759 (3)	C10—H10B	0.9600
C2—C3	1.376 (4)	C10—H10C	0.9600
C2—H2	0.9300	C11—N1	1.483 (3)
C3—C4	1.375 (4)	C11—C12	1.511 (4)
C3—H3	0.9300	C11—H11A	0.9700
C4—C5	1.380 (3)	C11—H11B	0.9700
C4—H4	0.9300	C12—C13	1.513 (4)
C5—C6	1.382 (3)	C12—H12A	0.9700
C5—H5	0.9300	C12—H12B	0.9700
C6—C7	1.470 (3)	C13—H13A	0.9600
C7—O3	1.344 (2)	C13—H13B	0.9600
C7—C8	1.346 (3)	C13—H13C	0.9600
C8—N1	1.441 (3)	N1—S1	1.6339 (19)
C8—C9	1.455 (3)	O1—S1	1.4268 (15)
C9—O4	1.219 (3)	O2—S1	1.425 (2)
C9—O5	1.327 (3)	O3—H3A	0.8200
			
C2—C1—C6	121.5 (2)	H10A—C10—H10C	109.5
C2—C1—S1	121.76 (19)	H10B—C10—H10C	109.5
C6—C1—S1	116.72 (17)	N1—C11—C12	114.2 (2)
C1—C2—C3	119.0 (2)	N1—C11—H11A	108.7
C1—C2—H2	120.5	C12—C11—H11A	108.7
C3—C2—H2	120.5	N1—C11—H11B	108.7
C4—C3—C2	120.5 (2)	C12—C11—H11B	108.7
C4—C3—H3	119.7	H11A—C11—H11B	107.6
C2—C3—H3	119.7	C11—C12—C13	111.4 (2)
C3—C4—C5	120.5 (3)	C11—C12—H12A	109.3
C3—C4—H4	119.8	C13—C12—H12A	109.3
C5—C4—H4	119.8	C11—C12—H12B	109.3
C4—C5—C6	120.4 (2)	C13—C12—H12B	109.3
C4—C5—H5	119.8	H12A—C12—H12B	108.0
C6—C5—H5	119.8	C12—C13—H13A	109.5
C5—C6—C1	118.1 (2)	C12—C13—H13B	109.5
C5—C6—C7	122.04 (19)	H13A—C13—H13B	109.5
C1—C6—C7	119.8 (2)	C12—C13—H13C	109.5
O3—C7—C8	123.2 (2)	H13A—C13—H13C	109.5
O3—C7—C6	113.3 (2)	H13B—C13—H13C	109.5
C8—C7—C6	123.46 (18)	C8—N1—C11	117.79 (16)
C7—C8—N1	120.99 (19)	C8—N1—S1	113.92 (15)
C7—C8—C9	120.03 (18)	C11—N1—S1	119.13 (14)
N1—C8—C9	118.98 (19)	C7—O3—H3A	109.5
O4—C9—O5	122.6 (2)	C9—O5—C10	115.99 (19)
O4—C9—C8	122.6 (2)	O2—S1—O1	119.30 (11)
O5—C9—C8	114.85 (18)	O2—S1—N1	108.17 (11)
O5—C10—H10A	109.5	O1—S1—N1	108.37 (10)
O5—C10—H10B	109.5	O2—S1—C1	107.40 (13)
H10A—C10—H10B	109.5	O1—S1—C1	109.72 (11)
O5—C10—H10C	109.5	N1—S1—C1	102.59 (9)
			
C6—C1—C2—C3	0.1 (4)	N1—C8—C9—O5	1.8 (3)
S1—C1—C2—C3	−178.3 (2)	N1—C11—C12—C13	176.1 (2)
C1—C2—C3—C4	−0.5 (4)	C7—C8—N1—C11	−108.9 (2)
C2—C3—C4—C5	0.9 (4)	C9—C8—N1—C11	72.3 (3)
C3—C4—C5—C6	−0.9 (4)	C7—C8—N1—S1	37.8 (3)
C4—C5—C6—C1	0.5 (3)	C9—C8—N1—S1	−141.04 (17)
C4—C5—C6—C7	−178.1 (2)	C12—C11—N1—C8	63.6 (3)
C2—C1—C6—C5	−0.1 (3)	C12—C11—N1—S1	−81.3 (2)
S1—C1—C6—C5	178.34 (18)	O4—C9—O5—C10	2.7 (3)
C2—C1—C6—C7	178.5 (2)	C8—C9—O5—C10	−177.5 (2)
S1—C1—C6—C7	−3.0 (3)	C8—N1—S1—O2	61.60 (18)
C5—C6—C7—O3	−21.8 (3)	C11—N1—S1—O2	−152.19 (18)
C1—C6—C7—O3	159.7 (2)	C8—N1—S1—O1	−167.73 (15)
C5—C6—C7—C8	158.4 (2)	C11—N1—S1—O1	−21.5 (2)
C1—C6—C7—C8	−20.2 (3)	C8—N1—S1—C1	−51.72 (16)
O3—C7—C8—N1	−177.71 (19)	C11—N1—S1—C1	94.49 (18)
C6—C7—C8—N1	2.1 (3)	C2—C1—S1—O2	100.0 (2)
O3—C7—C8—C9	1.1 (3)	C6—C1—S1—O2	−78.46 (19)
C6—C7—C8—C9	−179.1 (2)	C2—C1—S1—O1	−31.1 (2)
C7—C8—C9—O4	2.6 (3)	C6—C1—S1—O1	150.46 (17)
N1—C8—C9—O4	−178.5 (2)	C2—C1—S1—N1	−146.1 (2)
C7—C8—C9—O5	−177.1 (2)	C6—C1—S1—N1	35.43 (19)

### Hydrogen-bond geometry (Å, °)

**Table:**

*D*—H···*A*	*D*—H	H···*A*	*D*···*A*	*D*—H···*A*
O3—H3A···O4	0.82	1.84	2.558 (2)	145
C3—H3···O2^i^	0.93	2.48	3.358 (3)	158

Symmetry codes: (i) −*x*+3/2, *y*, *z*−1/2.
